# Explanatory models of depression in sub-Saharan Africa: Synthesis of qualitative evidence

**DOI:** 10.1016/j.socscimed.2019.112760

**Published:** 2020-02

**Authors:** Rosie Mayston, Souci Frissa, Bethlehem Tekola, Charlotte Hanlon, Martin Prince, Abebaw Fekadu

**Affiliations:** a Global Health and Social Medicine/King's Global Health Institute, King's College London, Social Science and Public Policy, NE Wing, Bush House, 30 Aldwych, London, WC2B 4BG, UK; b Institute of Psychiatry, Psychology & Neuroscience (IoPPN), Health Service & Population Research, King's College London, UK; c CDT-Africa, Main Library Building, College of Health Sciences, Addis Ababa University, PO Box 9086, Addis Ababa, Ethiopia; d Global Health & Infection Department, Brighton and Sussex Medical School, Brighton, UK

**Keywords:** Depression, Explanatory models, Qualitative synthesis, Sub-Saharan Africa

## Abstract

Debate about the cross-cultural relevance of depression has been central to cross-cultural psychiatry and global mental health. Although there is now a wealth of evidence pertaining to symptoms across different cultural settings, the role of the health system in addressing these problems remains contentious. Depression is undetected among people attending health facilities. We carried out a thematic synthesis of qualitative evidence published in the scientific literature from sub-Saharan Africa to understand how depression is debated, deployed and described. No date limits were set for inclusion of articles. Our results included 23 studies carried out in communities, among people living with HIV, attendees of primary healthcare and with healthcare workers and traditional healers. Included studies were carried out between 1995 and 2018. In most cases, depression was differentiated from ‘madness’ and seen to have its roots in social adversity, predominantly economic and relationship problems, sometimes entangled with HIV. Participants described the alienation that resulted from depression and a range of self-help and community resources utilised to combat this isolation. Both spiritual and biomedical causes, and treatment, were considered when symptoms were very severe and/or other possibilities had been considered and discarded. Context shaped narratives: people already engaged with the health system for another illness such as HIV were more likely to describe their depression in biomedical terms. Resolution of depression focussed upon remaking the life world, bringing the individual back to familiar rhythms, whether this was through the mechanism of encouraging socialisation, prayer, spiritual healing or biomedical treatment. Our findings suggest that it is essential that practitioners and researchers are fluent in local conceptualisations and aware of local resources to address depression. Design of interventions offered within the health system that are attuned to this are likely to be welcomed as an option among other resources available to people living with depression.

## Highlights

• Depression was perceived as problem with its roots in social adversity.
• Context matters-those engaged with the health system adopted biomedical models.
• Self-help and community resources were the first line in addressing depression.
• Traditional and biomedical medicine were used when symptoms were chronic or severe.
• Stepped care interventions should take into account local resources and models.

## 1 Introduction

Global mental health is about addressing inequities, namely the “treatment gap” for people with mental health conditions (Ndyanabangi et al., 2004; Roberts et al., 2018): the ethical imperative to provide access to scientifically evidence-based treatment for mental disorders, regardless of the socioeconomic context in which people live (Collins et al., 2011). Although studies suggest that the prevalence of depression in Low and Middle Income Countries (LMIC) and Sub-Saharan African (SSA) countries is comparable to that found in High Income Countries (HIC), at around 10–20 percent of the population at any one time; most people living with depression go untreated (Fekadu et al., 2017). Despite research carried out in primary healthcare settings indicating a high prevalence of depression among facility attendees, particularly in the context of co-morbid chronic disease (Ambaw, Mayston et al. 2015, 2017; Chibanda et al., 2016), detection rates remain low, including in contexts where clinicians have received training in detection and treatment of common mental disorders (Udedi, 2014; Fekadu et al., 2017; Rathod et al., 2018). Thus, a key objective for global mental health is to make sense of these discrepant findings. Critics of global mental health have suggested that psychiatric diagnostic criteria and accompanying standardised instruments, originated, developed and tested in the West are the source of artefactual findings related to the prevalence of depression in different cultural settings, and that the etic approach is invalid, given the role of the social and the cultural context in shaping the lifeworld (Littlewood, 1990; Patel et al., 1995a,b). Nonetheless, there is a range of evidence that indicates the credibility of common underlying concepts of mental sickness (Patel et al., 1997), with explanatory models indicating that around the world people experience depression through the body as well as the mind (Patel, 1995; William and Healy, 2001). For example, in their recent systematic review of qualitative evidence, Haroz et al. found that depressed mood/sadness; fatigue/loss of energy and problems with sleep were the three most common features of depression mentioned by participants in studies carried out among non-Western populations and Western populations (Haroz et al., 2017). Despite these commonalities, Haroz et al. highlight the potential limitations of using DSM-5 criteria. Social isolation, crying, anger and general pain were commonly mentioned features not included in DSM. Problems with concentration, psychomotor agitation or slowing (included in DSM) were infrequently reported. Haroz et al. point out the risk of tautological error, inherent to their search strategy, which used terms from the biomedical, psychiatric paradigm.

Our work aims to synthesise studies which examine the explanatory models of depression among people living in SSA (Kleinman et al., 1978). Our position accepts that the category of depression has become reified by researchers, clinicians and community members (Golden and Rosenberg, 1989; Kirmayer et al., 2017). We therefore anticipated that depression, and related concepts would be situated somewhere between disease and sickness, spiritual disturbance or life problem (Kleinman et al., 1978). We were interested to explore how depression was debated, deployed, described and managed in this anticipated context of contested meanings. We used explanatory models as a starting point for thinking about the aspects of depression most relevant to the central question of lack of detection of depression in SSA settings. This work was carried out as part of the Improving detection of depression in primary care in Sub-Saharan Africa- IDEAS Study, led by Prof. Abebaw Fekadu (African Research Leader scheme, Medical Research Council, UK).

## 2 Methods

We carried out a thematic synthesis of studies exploring explanatory models of depression among people living in sub-Saharan African (SSA) countries. Although the category of SSA is itself rightly contested, in this case, we felt that similarities in culture and society, including systems of healing, justified the category. We felt it was unlikely that we would identify enough evidence from particular regions or countries to be able to narrow this further. Our search strategy was pre-planned and designed to be comprehensive, according to inclusion criteria.

We included studies that were either wholly qualitative or included a qualitative component including In-depth interviews (IDIs); unstructured or semi-structured interviews; Focus Group Discussions (FGDs); free-listing/pile-sorts; participant observation; ethnography; or any combination of the above, where results of these activities were reported. Studies were excluded if they only reported quantitative data or if they were reviews, systematic reviews, editorials, notes or commentaries. Papers were included that reported results of primary research about depression carried out with people in community settings and community leaders, people living with depression, their caregivers, traditional or religious healers, or healthcare workers. Papers were eligible for inclusion if they reported findings carried out among adult participants (over 18 years old). Studies carried out among children (those younger than 18 years) were excluded, as were studies that explicitly addressed depression among pregnant women or depression during the postnatal period. Studies that addressed explanatory models of a generic notion of “mental illness” with no discrete focus on low mood, were excluded, as were studies which were carried out with a primary focus upon trauma and post-traumatic stress disorder.

We searched the following databases Medline; Embase, PsychInfo and Global Health, as well as searching the reference lists of all included studies and consulting with experts in the field. We combined the following sets of search terms: “Depression” OR “depressive disorder” OR “melancholia” OR “depressive disorder, major” AND “anthropolog*” OR “qualitative” OR “ethnograph*” OR “cross-cult*” OR “ethnopsychol*” OR “cultur*” OR “phenomenonlog*” OR “idioms” OR “percept*” OR “beliefs” OR “understand*” AND list of SSA countries, separated by OR and including “Africa”, “Sub-Saharan Africa”. We included only studies with abstracts published in English.

Study screening was carried out in two stages. First, title and abstract of search results were independently reviewed by RM. Second, full-text were independently reviewed by RM and SF Results were discussed with AF, who resolved any disagreements between RM and SF.

The CASP (Critical Appraisal Skills Programme) checklist for qualitative studies was used to guide critical appraisal of studies deemed to be eligible according to inclusion and exclusion criteria. Studies were included if they presented sufficient information about the methods used and the context; in particular, appropriate research design and methodologies to address the stated research question, including the extent to which description of methods suggests that results remain grounded in the experience of participants; recruitment procedures well described; ethical considerations discussed. It was decided not to exclude studies based on insufficient description of reflexivity and the relationship between researcher and participant. This was seldom discussed. Likewise, few studies discussed data analysis approach and procedures in detail.

A data extraction form designed by RM was used: this included background information about each of the studies (authors, year, country, methodological approach, participants-including sampling/recruitment strategy, inclusion/exclusion criteria, research questions/aims, data collection techniques, data analysis approach, results-main themes, strengths and limitations identified by authors, reviewer comments on quality). Results and discussion were extracted for analysis. Analysis was carried out using OpenCode 4.0, an open-source software for analysis of qualitative data (Umeå, 2013). We were guided by the approach set out by Thomas et al. describing thematic synthesis for systematic reviews of qualitative research, which recommends coding and synthesis is organised in three stages (Thomas and Harden, 2008). Firstly, text was coded line-by-line by RM, using descriptive codes, with codes added incrementally to a “bank” as new text was coded and it became necessary to introduce new codes. Secondly, codes were grouped together thematically, to capture meaning, and a hierarchical structure was introduced. For example-one thematic group was “emotional problems”, which collected together descriptions of sadness, negative/low mood and anger. A final level of descriptive coding was added, grouping together related themes, eg. “emotional problems”, “thinking too much” and “problematic behaviours” were arranged under “characterising the problem”, resulting in a tree-like coding framework. The third and final stage of coding involved moving beyond description of the content of the original studies to generation of “third order interpretations”. We used a version of the explanatory models framework, expanded beyond the domains described by Kleinman in 1978 (Kleinman et al., 1978) to include: aetiology, onset of symptoms, pathophysiology, course of sickness and treatment to include ideas about help-seeking and help-seeking behaviours, beliefs about health and illness, effect of the problem (upon body, emotions, social life/relationships, economic status) (Lloyd et al., 1998). Our aim was to ensure an inclusive approach which was likely to capture any aspect of an individual's beliefs, meaning attached to illness, expectations about what will happen to them/a person with the problem which the individual deems important enough to include in their narrative explanation of illness. Our coding framework was compared against the explanatory model framework and codes were grouped according to themes within the explanatory model.. Coded text was considered within these categories, links across different studies were explored, with the aim of understanding underlying commonalities, for example, shared concepts and beliefs across different studies and different country settings. Simultaneously, links between thematic categories were explored, for example, how signs and symptoms might be connected to ideas about causality, which, in turn, might shape approaches to seeking help. Ultimately, these relationships were considered in light of what they were able to tell us about conceptualisation of depression-like illness in the SSA region. We ensured grounding of analysis in the context in which data were originally constructed by consideration of coded text alongside the background information we had extracted for each study.

## 3 Results

### 3.1 Description of studies

A total of 25 studies were included (see Table 1). Ten studies were carried out in Southern Africa (South Africa (Sorsdahl et al., 2010; Petersen et al., 2013; Burgess and Campbell, 2014; Andersen et al., 2015; Johnson et al., 2017a,b; Myers et al., 2018), Zambia (Aidoo and Harpham, 2001), Zimbabwe (Patel et al., 1995a,b; Patel et al., 1995a,b; Kidia et al., 2015)), 14 were carried out in East Africa (Ethiopia (Monteiro and Balogun, 2014), Rwanda (Bolton, 2001), Burundi (Familiar et al., 2013; Ventevogel et al., 2013; Irankunda and Heatherington, 2017; Irankunda et al., 2017), South Sudan (Ventevogel et al., 2013), Uganda (Wilk and Bolton, 2002; Okello and Ekblad, 2006; Okello and Neema, 2007; Johnson et al., 2009; Okello et al., 2012; Nakimuli-Mpungu et al., 2014; Johnson et al., 2017a,b; Murray et al., 2017)); one was carried out in central Africa (Democratic Republic of Congo) (Ventevogel et al., 2013) and one was carried out in West Africa (Ghana) (Psaki and Hindin, 2016). Six studies were carried out in a post-conflict setting (Burundi, Democratic Republic of Congo, Rwanda, South Sudan, northern Uganda). Ten studies were carried out among clinical populations; including studies carried out among people living with HIV (N = 6: Southern Africa (Petersen et al., 2013, Andersen et al., 2015; Kidia et al., 2015; Myers et al., 2018); Uganda (Okello et al., 2012; Murray et al., 2017)); people receiving treatment for depression (N = 5: Uganda (Okello et al., 2012; Nakimuli-Mpungu et al., 2014; Johnson et al., 2017a,b; Johnson et al., 2017a,b); Zimbabwe (Patel et al., 1995a,b)) and people attending primary healthcare clinics where there was no mental health service (N = 2: Burundi (Irankunda and Heatherington, 2017; Irankunda et al., 2017)). We identified eight studies that took a completely open-ended approach, carried out among community samples (without screening or diagnosis) (Patel et al., 1995a,b; Bolton, 2001; Wilk and Bolton, 2002; Familiar et al., 2013; Ventevogel et al., 2013, Monteiro and Balogun, 2014); participants in these studies were asked to list and describe problems in their communities. Six studies used vignettes to describe depression (Patel et al., 1995a,b; Okello and Ekblad, 2006; Johnson et al., 2009; Sorsdahl et al., 2010; Psaki and Hindin, 2016; Johnson et al., 2017a,b). Six studies used Kleinman's Explanatory Models questions as the basis for interviews (Patel et al., 1995a,b; Okello and Ekblad, 2006; Johnson et al., 2009; Johnson et al., 2017a,b; Johnson et al., 2017a,b). Eleven studies included people with either a clinician diagnosis of depression or a positive screen for depressive symptoms using a standardised measure (Primary Care Checklist, Centre for Epidemiological Studies Depression Scale- CES-D, Beck Depression Inventory, Self-Report Questionnaire- SRQ, Hopkins Symptom Checklist, Shona Symptom Questionnaire) (Aidoo and Harpham, 2001; Okello and Neema, 2007; Okello et al., 2012; Petersen et al., 2013; Nakimuli-Mpungu et al., 2014; Andersen et al., 2015; Kidia et al., 2015; Johnson et al., 2017a,b; Johnson et al., 2017a,b; Murray et al., 2017; Myers et al., 2018). Eight studies included samples of community members (Patel et al., 1995a,b; Bolton, 2001; Wilk and Bolton, 2002; Okello and Ekblad, 2006; Johnson et al., 2009; Familiar et al., 2013; Psaki and Hindin, 2016; Johnson et al., 2017a,b), including one study which was nested in a community survey: participants completed depression, anxiety and Post-Traumatic Stress Disorder screening tools which were referenced in analyses but not used in recruitment (Burgess and Campbell, 2014). Six studies included traditional or faith healers (Patel et al., 1995a,b; Okello and Ekblad, 2006; Sorsdahl et al., 2010, Monteiro and Balogun, 2014, Psaki and Hindin, 2016, Johnson et al., 2017a,b) and six studies included healthcare workers in their samples (Patel et al., 1995a,b; Aidoo and Harpham, 2001; Johnson et al., 2009; Monteiro and Balogun, 2014; Johnson et al., 2017a,b; Johnson et al., 2017a,b). Twelve studies used semi-structured interviews (Patel et al., 1995a,b; Aidoo and Harpham, 2001; Okello and Neema, 2007; Johnson et al., 2009; Okello et al., 2012; Familiar et al., 2013; Monteiro and Balogun, 2014; Irankunda and Heatherington, 2017; Irankunda et al., 2017; Johnson et al., 2017a,b; Johnson et al., 2017a,b; Murray et al., 2017), seven studies used in-depth interviews (Okello and Ekblad, 2006; Petersen et al., 2013; Burgess and Campbell, 2014; Andersen et al., 2015; Kidia et al., 2015; Psaki and Hindin, 2016; Myers et al., 2018) and six studies used FGDs (Patel et al., 1995a,b; Okello and Ekblad, 2006; Sorsdahl et al., 2010; Nakimuli-Mpungu et al., 2014, Psaki and Hindin, 2016); three studies used free-listing and/or pile sorts (Bolton, 2001; Wilk and Bolton, 2002; Familiar et al., 2013). Six studies used a mixture of these different methods (Bolton, 2001; Wilk and Bolton, 2002; Okello and Ekblad, 2006; Familiar et al., 2013; Ventevogel et al., 2013; Psaki and Hindin, 2016; Irankunda and Heatherington, 2017).

**Table 1:** 

#	Author/Year	Country	Methodological approach	Participants	Research questions/aims	Main themes
1	Aidoo and Harpham, 2001	Zambia	Qualitative interviews using modified version of Kleinman's EM questions; group interviews with HCWs	Household survey in Mtendere, Lusaka (n = 323) identifying 139 women with depression (scoring >7 on SRQ) 10 HCWs providing MH services to the Mtendere community- 6 x psychiatrists, 1 x psychologist and 3 x social workers (urban Lusaka).	To compare the EMs of HCWs and low income urban women from the community in which they work	Name given to women's experience; cause of stress; onset of stress; how the experiences of stress make women feel; severity of stress; women's greatest fears about their experience of stress; choice of treatment; influences leading to choice of treatment; results hoped to be received from treatment
2	Andersen et al. (2015)	South Africa	Informed by grounded theory, in-depth interviews, open coding followed by axial and selective coding	14 adult attendees of an infectious disease clinic in a township east of Cape Town with a diagnosis of MDD (using the MINI)	To describe the experience of black South Africans living with HIV and depression	Symptoms: affective, cognitive, behavioural; relevance of HIV to depression symptoms; depression treatment history
3	Bolton (2001)	Rwanda	Ethnographic methods: a) free-listing of MH symptoms and disorders; b) informant interviews; c) pile sorts to confirm relationships between symptoms/disorders	Residents of 2 x rural administrative regions near Kigali: a) 41 people judged to be knowledgeable about community problems by interviewers; b) people identified in a) as individuals people would consult about MH problems; c) convenience sample (n = 40)	To investigate how Rwandans perceive the mental health effects of the 194 genocide; to investigate the local validity of western mental illness concepts; to provide data to adapt existing MH assessments for local use	*Guhahamuka* and *agahinda* include all the symptoms categories required for DSM-IV diagnoses of PTSD and depression
4	Burgess and Campbell, 2014	South Africa	In-depth interviews, grounded thematic analysis	19 women, living in Umkhankude sub-district, in North Kwa Zulu Natal province. Randomly selected from list of participants in a community survey about social deprivation and HIV. Participants completed CES-D (depression), Beck Anxiety Inventory, Harvard Trauma Questionnaire but were not selected on basis of scores.	To obtain a fine-grained account from women about perceived links between HIV and MH; to explore women’s understanding of mental distress among a non-clinical population; to understand women’s’ efforts to cope	Women were economically dependent upon men: their distress was often grounded in abandonment and impoverishment. Conflict in other relationships, HIV and violence were also important drivers of distress. Coping often involved psychological reframing of life struggles towards acceptance. Government grants enabled women to survive and income generation opportunities were sought. These strategies rarely led to long-term resolution.
5	Familiar et al. (2013)	Burundi	a)Free-listing and b) semi-structured interviews with key informants	Residents in 5 communities in the Kibuye Health District (high population density area) purposive sampling: a) n = 38; b) n = 23	To explore community perceptions of mental distress, understand key concepts and associated behaviours to inform future service delivery and policy development	*Ukutiyemera* corresponds to DSM-IV description of dysthemia and depressive disorder. Traditional healer, priests, community health centres were all potential sources of help. However, HCWs reported that many remain at home without treatment. Most important impact of mental distress was loss of assets.
6	Irankunda and Heatherington, 2017	Burundi	Mixed methods: semi-structured interviews- asking about treatment preference for three conditions (quantitative) and rationale for treatment expectations (qualitative). Constant comparison involving three researchers	N = 198 attendees of primary care at the Village Health Works clinic, Kigutu (service run by NGO- no mental health screening as no mental health services available; rural)	To describe expectations of efficacy of four different treatments (spiritual, traditional, medication, evidence-based psychosocial interventions) across three key syndromes, including *akabonge* (depression-like symptoms)	Explanations for why the four treatment options would/would not work
7	Irankunda and Heatherington (2017)	Burundi	a)pilot study- open ended group discussions; b) semi-structured interviews asking about three syndromes identified in a) and symptoms/causes of these; c) semi-structured interviews presented syndromes identified in b) and asking what they would be called	a) n = 761 (14 groups, ranging from 25 to 50 participants); b) n = 542 (52% men); c) n = 143 (46% men). Attendees of the Village Health Works clinic, Kigutu (service run by NGO- no mental health screening as no mental health services available; rural). a) was conducted as part of the public health discussions held with people in the waiting room each morning (*education pour la sante*).	To derive a basic taxonomy and description about mental health problems.	Evidence of three major conditions: the closely related conditions of *kuyinga* and *akabonge*/i*bonge*; *guhahamuka* and *gusara.* Partial overlap with depression, PTSD and schizophrenia, respectively. Causes of *akabonge* include: war, problems, loss of loved ones, sadness, loneliness
8	Johnson et al. (2009)	Uganda	Semi-structured interviews based on Kleinman's EM questions	Residents of Kampala or surrounding peri-urban districts: community members (n = 135); HCWs (n = 111) (from 44 districts- recruited from markets, businesses, health services).	To compare EMs between community members and the HCWs who provide a service in that community	Contextual relevance of depression; labelling and conceptualisation; aetiology; impact and social meanings; help-seeking; type of treatment; treatment expectations; difference based on social characteristics
9	Johnson et al., 2017a	Uganda	Semi-structured interviews based on Kleinman's EM questions	Residents of Kampala or surrounding peri-urban districts: community members (n = 135); HCWs (n = 111) (from 44 districts- recruited from markets, businesses, health services); people meeting criteria for depression and seeking therapy services (n = 33, from 17 districts)	To explore whether EMs predicted help-seeking through assessment of the relationship between problem conceptualisation and treatment	Problem conceptualisation was not a predictor of treatment choice among either community members, HCWs or the sample of people with depression.
10	Johnson et al., 2017a,b	Uganda	Semi-structured interviews based on Kleinman's EM questions	Adult patients at traditional healing and psychiatry clinics (n = 30) and patient-provider dyads (n = 8) near Kampala, Uganda	To investigate differences in EMs associated with help-seeking (traditional or psychiatric services) among patients and providers	Patients in both settings had similar EMs in terms of symptoms, perceived cause, seriousness, impact of depression. However, those attending traditional clinics had a preference for herbal treatments, whilst patients at psychiatric clinics were more likely to desire biomedical treatment.
11	Kidia et al. (2015)	Zimbabwe	In-depth interviews	Purposive sample of attendees at the HIV clinic of Parirenyatwa Hospital, Harare (one of the largest treatment facilities in the country) who were living with HIV and scored >5 on the Shona version of the SRQ-8 (n = 47)	To explore lived experience of adults living with HIV and co-morbid CMD with poor adherence to ART in order to develop a culturally appropriate intervention	Challenges- poverty, stigma, marital problems, symptoms of CMDs; impact of challenges on adherence and access to ART: poverty, stigma; intervention ideas: family engagement and disclosure, income generation and transport, privacy
12	Monteiro & Balogun 2014	Ethiopia	Semi-structured interviews	Purposive sampling of HCWs (n = 35), lay people (n = 75) and traditional healers (n = 5) resident in Addis Ababa (n = 82) and Asella, rural Southern Ethiopia (n = 33).	To explore ideas about the definition and expression, causation and treatment of mental illness and perceptions of depression, anxiety and psychosis? To examine whether community members/laypersons, healthcare workers and traditional healers differ in their attitudes, beliefs and practices regarding mental illness	Participants from all three groups agreed that you would know someone had depression from their negative affect/emotions; the most common cause of depression identified was loss of loved one/death. HCWs and lay people felt that advice and counselling and social support were the most appropriate treatment; where as traditional healers thought opted for traditional/cultural treatments
13	Murray et al. (2017)	Uganda	Semi-structured interviews	Purposive sample (n = 9), those scoring > mean on Hopkins Symptom Checklist- caregivers of young children living with HIV, participants in the control arm of an RCT (n = 60) of parenting intervention- attendees of local clinics/NGOs providing HIV care in Tororo and Busia district, Eastern Uganda (rural).	To create a contextually embedded conceptual framework of the relationship between caregiver mental health and HIV-infected child well-being, in order to inform support services for families living with HIV	Fulfilling the caregiving role; how caregiver mental health affects children; how child sickness affects caregivers' lives; mental health and inability to provide; duality of support and isolation
14	Myers et al. (2018)	South Africa	Qualitative interviews	Maximum variation sampling of patients attending 17 Primary Healthcare Clinics receiving care for diabetes (n = 11) or HIV (n = 19) in the Western Cape (n = 30, includes n = 1 co-morbid diabetes and HIV); scoring >16 on the CES-D or >8 < 22 on the AUDIT (for Alcohol Use Disorder). “Almost all” scored >16 on CES-D.	To describe patients living with chronic disease perceptions of acceptability of mental health counselling in the context of PHC as well as preferences for mode of delivery of counselling	Screening for mental health problems was felt to be useful as poor awareness meant people wouldn't proactively seek help. Coping with stress, often linked to chronic disease diagnosis was a key problem/need. Brief counselling was preferred, delivered by specialist mental health counsellor
15	Okello and Ekblad, 2006	Uganda	a) focus group discussion, c) in-depth interviews with key informants using case vignettes. Constructivist version of grounded theory was selected as frame of analysis	Community members from Bajjo, small village in Mukuno (23 km from Kampala, semi-rural). a) (n = 5) traditional and faith healers; b) (n = 25, of which n = 13 women) 4 groups: secondary school girls; women (mean age 35yrs); men (mean age = 38yrs); primary school teachers (mean age = 35yrs); c)	To assess the feasibility of using case vignettes to explore local explanatory models for various sub-types of depressive illness- including aetiological factors, perceived effects of depressive symptoms and appropriate forms of help	Identity given to symptoms according to type of depression; aetiological factors associated with onset of depression- psychological factors, socioeconomic, spiritual/cultural factors, biological/physical factors; effects of depressive symptoms; sources of care for depression
16	Okello and Neema, 2007	Uganda	Semi-structured interviews based on Kleinman's EM questions. Analysis was informed by symbolic interaction theory- “all objects, events, situations acquire their meaning through a process of human interpretation”	Purposive sample of people with an Axis I depression diagnosis accessing a mental health clinic at Mulago Hospital, Kampala (n = 22) (national referral hospital, regional hospital for the central region)- referred to researchers by psychiatrists working at the clinic. Clinician diagnosis confirmed by researcher using MINI.	To explore how people diagnosed with depression conceptualised their illness and how the conceptualisation shaped courses of action in the search for help. Intention was to capture the complexity of the decision to seek help	Somatization, social meaning of depression and help-seeking; meaning and perceived consequences of illness; “How did I get here”- making sense of psychiatric admission; variations in the causal attribution and the role of significant others in help-seeking
17	Okello et al. (2012)	Uganda	Semi-structured interviews, thematic analysis	Consecutive attendees receiving ART at a large HIV treatment centre (caseload n = 11000), diagnosed with major depressive disorder by clinic psychiatrist (DSM IV) (n = 26, n = 11 had received antidepressants treatment, n = 15 interviewed before starting antidepressants)	To explore how depressed people living with HIV in Uganda conceptualised and described their depression and its manifestation in the context of ART and anti-depressant treatment	Depression symptoms reported by participants; attributions of depression in the context of ART; the effects of ART alone on the psychological health of clinically depressed individuals; the effects of antidepressants on the psychological wellbeing of clinically depressed ART patients
18	Nakimuli-Mpungu et al., 2014	Uganda	Focus groups, thematic analysis	Attendees of Peter C. Alderman Foundation (PCAF) trauma clinics in Gulu and Kitgum, northern Uganda (areas that have experienced >20 years of civil war) who were receiving treatment for depression/had experienced depression in the past (identified from clinic records by staff) and their caregivers (n = 110).	To obtain information on the cultural understanding of depression symptoms, complications and treatment methods used in post-conflict communities in northern Uganda, in order to inform the development of an indigenous group support intervention to treat depression	Community perceptions of depression and mental health problems; community strategies used to combat depression in the acholi community; community perceptions of counselling; structure and content of group support intervention;
19	Patel et al. (1995)	Zimbabwe	Focus groups	N = 9 Focus Group Discussions (FGDs) in total- participants were selected by investigators, who had existing links with the relevant stakeholder groups: n = 30 village community workers took part in n = 3 FGDs; n = 22 traditional and faith healers took part in n = 3 FGDs; n = 9 community psychiatric nurses took part in a FGD and n = 15 relatives of patients attending out-patient clinics at the Parirenyatwa Hospital, Harare took part in n = 2 FGDs	To generate information on the concepts of mental illness from a range of care-providers in Harare	Symptoms; impact; sources of care
20	Patel et al. (1995)	Zimbabwe	Semi-structured interviews based on EMIC followed by CISR	N = 109 attendees of primary care at three health centres and the clinics of four traditional healers in high density suburbs of Harare	To elucidate symptom profiles and describe explanatory models of illness among people considered to have mental illness by care providers	Somatic complaints were the most common presentation but only one fifth of patients thought that their illness was purely one of the body. Illness was chronic- this is likely to reflect bias of providers. Spiritual causes were the most frequently mentioned explanation, with “*kufungisa*”- thinking too much identified as a proximate cause.
21	Psaki and Hindin, 2016	Ghana	Mixed methods to examine the validity of the CES-D scale, nested in Family Health and Wealth Study (FHWS- multi-country cohort study) including: a) focus groups and b) In-depth interviews; inductive content analysis	a) n = 12 FGDs, with a total of n = 95 participants (42 men and 53 women) who were FHWS participants; b) n = 19 religious leaders, healthcare providers, community elders/leaders (11 men, 8 women, not FHWS participants)	To use mixed methods to assess whether the CES-D 10 scale effectively captures culturally relevant domains of depressive symptoms among men and women of reproductive age in Kumasi, Ghana.	Most common symptoms associated with depression were: loss of concentration, crying, loss of appetite, becoming quiet or withdrawn. Suicide was a common impact of depression
22	Petersen et al. (2013)	South Africa	In-depth interviews based on Kleinman's EMs approach. Thematic content analysis- a priori plus emerging codes, constant comparison	N = 35 women living with HIV and depression, attendees of HIV/AIDS two clinics in KwaZulu-Natal and North-West Province (peri-urban). Participants were those who attended either clinic on 4 randomly selected days who scored >8 on the SRQ-20 and were diagnosed with major depressive disorder using the Structured Clinical Interview for DSM-IV Diagnosis (SCID). Excluded if pregnant/delivered baby in last 6 months.	To understand the context and local understandings of depression among women living with HIV, with a view to informing the content of a culturally acceptable care package for depression; to develop an understanding of how best to deliver this package using a collaborative care task-sharing approach within existing resource constraints	Perceived causes of depressive symptoms and exacerbating factors: being HIV-positive, stigma & discrimination, lack of social support, partner rejection/abuse/abandonment, other family problems, poverty related stress, trauma and loss; depressive symptoms; current coping strategies and possible interventions
23	Sorsdahl et al., 2010	South Africa	Focus groups using vignettes from the Short Explanatory Model Interview (SEMI); in-depth interviews. Framework approach to analysis	Convenience sample of n = 50 (4 FGDs with 8 healers in each group and 18 in-depth interviews). Traditional healers were selected from those who attended a workshop conducted by the South African Depression and Anxiety Group (mental health advocacy group) in Mpumalanga.	To identify concepts, causes, and treatments for mental disorders among traditional healers, including exploring basic concepts and contrasting responses to a psychotic vignette with responses to vignettes representing non-psychotic mental disorders	Healers did not think case described in depression vignette was a mental illness but was a problem caused by psychological reasons. Those who thought the patient was suffering from an illness (68%) believed this required attention of a traditional healer (40%) or a western doctor (28%).
24	Ventevogel et al. (2013)	Burundi, Democratic Republic of Congo, South Sudan	“Rapid ethnographic assessment”- Focus Group Discussions (FGDs) and key informant (KI) interviews. FGD ad KI participants were asked to talk about “problems or illness that manifest through problems in thinking, feeling or behaving”. KI participants were also asked to talk about their work. Thematic analysis.	n = 31 FGDs with a total of n = 251 participants, across four broadly agriculture-focussed rural sites affected by conflict: Kwajena Payam and Yei (South Sudan); Butembo (DRC); Kibuye (Burundi). Separate FGDs were held for men and women. The aim was to achieve sample characteristics broadly consistent with site demographics. N = 26 KI interviews were held with local experts on mental health problems- traditional/religious healers, healthcare workers and policy makers	To explore local concepts of mental disorder in four settings in Africa	Identification of five syndromes with similarities to depression/anxiety (South Sudan- *yeyeesi.* “people whose mind is busy with thoughts”, DRC- *Amutwe alluhire* “tired head”, Burundi- *ibonge/akabonge*- dwelling on loss, South Sudan- *nger yer* “cramped stomach, always sad”. Causes were perceived to be loss/worry- including of livelihood and properties but particularly loved ones. Social interventions to provide support and to address isolation were recommended by participants.
25	Wilk and Bolton, 2002	Uganda	Free-listing and key informant interviews	Free-listing participants were 50 local people from 10 villages. Participants were asked “what are the main problems that affect the people of this community as a result of HIV?“. Twenty key informants (people whom local consultants identified as being knowledgeable about mental health). Interviews were focussed on problems identified in free-listing.	To understand how people perceive the mental health effects of HIV, including examining the validity of western concepts of PTSD and depression.	Free-listing identified eight psychological problems from a total of 30 problems reported. Two syndromes were identified “*Yo'kwekyawa*”- “hating oneself” and “*Okwekubaziga*”- “pitying oneself”, which, between them, include all of the nine major DSM-IV depression criteria.

### 3.2 Naming and describing the problem

Studies that asked participants to list problems in their community found that depression-like illnesses were one problem among many, ranked below economic problems. Studies that adopted an open-ended approach identified three main types of problem/illness similar in character to depression. A deep sadness condition, characterised by negative affect (Monteiro and Balogun, 2014): “*agahinda*” or “*intuntu*” (Burundi and Rwanda) (Bolton, 2001; Irankunda et al., 2017); “owekubaziga”(Wilk and Bolton, 2002) or “*obunakuwavu*” (Johnson et al., 2017a,b) (Uganda)- can develop into a more serious problem, eg. “*akabonge/kuyinga*” (Burundi), attributed to loss of loved ones and associated mainly with affective symptoms but some somatic (chest and head pain) (Familiar et al., 2013; Ventevogel et al., 2013; Irankunda et al., 2017), similar to “*yo'kwekyawa*” (Uganda) (Wilk and Bolton, 2002). In Democratic Republic of Congo, *“amutwe alluhire”*, ‘tired heads’ included many of the symptoms of major depression, whilst in South Sudan, *“nger yec”* included all of the core DSM symptoms of depression, apart from excessive guilt, but was distinguished as a specific syndrome by participants by stomach pain and diarrhoea:

“When a father dies and he has three sons, all will cry. But one son cries too much. That one has nger yec. He feels it in his stomach. Sometimes a person can even tie his belly with a rope to stop the cramp.” (FGD, Kwajena, South Sudan) (p9) (Ventevogel et al., 2013)

“*Ukutiyemara*” (Burundi) (Familiar et al., 2013) and “*yeyeesi*” (South Sudan) (Ventevogel et al., 2013) shared many of the symptoms identified as “*kufungisa*” (Zimbabwe) (Patel et al., 1995a,b) and “*okweraliikirira*” (Uganda) (Okello and Ekblad, 2006); most prominently, “thinking too much” (“*kwelalikilira*” (Johnson et al., 2017a,b) (Uganda). “Thinking too much” was identified as a key feature of depression in all the studies carried out among people living with HIV (Okello et al., 2012; Petersen et al., 2013; Andersen et al., 2015; Kidia et al., 2015; Murray et al., 2017). In the context of HIV, worrisome thoughts were particularly related to the impact of HIV status on family members (Petersen et al., 2013).

In twenty-one studies, the conditions described were seen as ‘mental disturbance’, “sickness of the soul” (Okello and Ekblad, 2006) or “burdened hearts” (Kidia et al., 2015) but differentiated from “madness”, which was perceived to be a more severe category, associated with disruptive behaviour, such as throwing stones, abusing people and/or running around naked (Okello and Ekblad, 2006; Ventevogel et al., 2013). An exception to this were participants in Okello et al.'s study, whose admission as psychiatric inpatients, according to the author, had forced them to realise that “they had a condition that is conventionally defined as mental illness” (p21) (Okello and Neema, 2007). In Buganda, as in many other societies, mental illness is highly stigmatised and therefore having a mental illness has significant implications for one's identity (Okello and Neema, 2007). Where they were compared, there was evidence of conceptual differences between healthcare workers and people living with the condition. For example, in Zambia, healthcare workers used the words “stress and depression” to describe a health problem, female patients differentiated between “problems of the mind” (low self-esteem, unhappiness, thoughts of suicide) and physical symptoms (head-aches, palpitations) (Aidoo and Harpham, 2001).

### 3.3 Causes

Difficult life circumstances were commonly perceived to be the overarching cause of the problems described (Patel et al., 1995a,b, Aidoo and Harpham, 2001, Sorsdahl et al., 2010, Okello et al., 2012, Ventevogel et al., 2013; Burgess and Campbell, 2014; Monteiro and Balogun, 2014, Irankunda and Heatherington, 2017, Irankunda et al., 2017, Johnson et al., 2017a,b). In some cases, specific experiences were linked to a particular problem, for example “akabonge” was associated with women who have lost a child (Familiar et al., 2013). However, psychological or social factors were often seen to be the proximate causes of the problem (Sorsdahl et al., 2010), for example, thinking too much about problems (Ventevogel et al., 2013), such as bereavement or relationship issues:

*“You go on combing thoughts; like us women, the thoughts may not necessarily be about jobs, say a friend comes and tells you ‘when your husband was returning home, he passed (branched off) to Nankya's place for evening tea’ Then you remember that he did not eat the food you gave him for supper. So that makes the women develop thoughts. This causes illness of thoughts”* (FGD women) (p299) (Okello and Ekblad, 2006).

The manifestation of poverty most commonly discussed was the impact upon children and concern about fulfilling their basic needs for food, accommodation and education (Okello and Ekblad, 2006; Petersen et al., 2013; Burgess and Campbell, 2014; Kidia et al., 2015; Murray et al., 2017):

*“Now my child has been sent back home from school. What am I supposed to do with the child? I started crying. I don't have anything to do. I don't go to work. Sometimes I wash clothes and sweep for people for money. But right now I don't* have *anything to do, not even selling sweets. My child is not going to school (ID #28, female, 44 years)* (p906) (Kidia et al., 2015)

Economic dependence on spouses meant that financial problems and relationship problems were perceived to be entangled (Patel et al., 1995a,b; Okello and Ekblad, 2006; Burgess and Campbell, 2014; Kidia et al., 2015). Women described choices about spending by husbands as an indication of the quality of their relationship, with betrayals expressed in economic terms:

*“We may be* patient *and work hard to get out of poverty but the time when we've got the money, when I would also be able to enjoy myself, my husband neglects me and goes out looking for another woman with big hips to enjoy life”* (FGD women) (p299) (Okello and Ekblad, 2006)

Male control, including the threat of violence and abuse was identified by women as a triggering factor for distress (Patel et al., 1995a,b; Petersen et al., 2013; Kidia et al., 2015). In one study carried out in Uganda, female focus group discussion participants described how the quality of a relationship was ultimately more important than economic status:

*“Sometimes having a good relationship in marriage is better than having money and misunderstanding every day. Money can be useless if you have marital problems...at least you would rather be with a husband when you are poor but you are on good terms”* (FGD women) (p300) (Okello and Ekblad, 2006)

HIV was perceived to exacerbate both economic and relationship problems. For example, disease-related stigma provided an explanation for spousal antagonism and shaped the language of conflict between partners:

*“What can I say [what causes my depression] I think it's because at home we are not getting along...he doesn't want to hear about it [my HIV status]...I told him to go for testing he doesn't want to even if I told him that I'm on ARVs he said that he doesn't care and said that he does not want to see these pills in the house...so I'm in this on my own.”* (Case 18, KZN) (p560) (Petersen et al., 2013)

Having a presumed seroconcordant male partner was linked to a parallel set of challenges, with women under pressure to share medication with husbands who were too embarrassed to attend clinics for testing or treatment (Kidia et al., 2015). Being HIV-infected aggravated economic problems: participants struggled to meet the costs of travelling to health services in the context of already stretched finances and competing priorities:

*“Money is a very big problem. Sometimes I have to sell a chicken, and they're almost finished. I have to sell two* chickens *for me to come here [the HIV clinic], 7 dollars each, to and fro”* (ID #31, Male, 37 years) (p907) (Kidia et al., 2015)

HIV was linked to poor physical health and not working, which, in turn, was linked to an intensification of worry (Burgess and Campbell, 2014):

*“But I keep on thinking and I am not sleeping. Then I also feel sick. I eat well but I want to be able to work so that I can* sustain *myself. I worry that I am not working…So every time I think about my not being able to go back [to work]; I start thinking too much and I begin to worry..”* (Participant 4) (p40) (Okello et al., 2012)

As well as being entangled with relationship problems, HIV-related stigma was perceived to have its own direct impact upon depressive symptoms:

*“HIV* causes *depression due to stigma. Other people ask you if you are struggling for something with them, they ask you, when will you die? How I wish you will die sooner”* (Male participant, Namukora, Kitgum district) (p13) (Nakimuli-Mpungu et al., 2014)

Anticipated stigma created pressure not to disclose HIV-status and was identified as another source of stress (Kidia et al., 2015). For some, HIV diagnosis and/or initiation of ART treatment were explicitly identified as either a direct cause (Wilk and Bolton, 2002, Johnson et al., 2017a,b) or starting point for depression (Okello et al., 2012; Andersen et al., 2015). Although ART improved physical health, symptoms of depression endured, perpetuating an inability to work (Okello et al., 2012). Findings from a South African study carried out among chronic disease clinic attendees suggested that adjustment to diagnoses for both HIV and diabetes were associated with stress and fatalism, which in turn was linked to a continuation of unhealthy habits such as drinking and smoking (Myers et al., 2018).

Spirits and witchcraft were discussed as a possible cause of depressive symptoms in some settings, particularly Uganda in studies conducted among non-HIV-affected populations (Okello and Ekblad, 2006; Okello and Neema, 2007; Nakimuli-Mpungu et al., 2014): for example, possession by evil spirits of ancestors having caused offence/upset by converting to Christianity or by failing to perform rituals associated with particular duties (Okello and Ekblad, 2006), or others, for example, killing someone during wartime (Nakimuli-Mpungu et al., 2014). Alternatively, bewitchment by somebody living, such as a co-wife, might occur because of jealousy (Nakimuli-Mpungu et al., 2014). Somatic, cognitive and affective symptoms were discussed alongside the social problems or disjuncture to which they were perceived to be connected. The patterning of these connections were suggestive of the likely cause:

*“My head has a problem. [Would you please elaborate?] My head has a problem it gets chaotic. Sometimes is like there bells ringing in it. I got fever, but you see in the village I am active. I have been involving myself in a number of activities. Even the local leader in* our *area had promise to take me a course in modern agriculture. People may not be happy about my progress. They become jealous of me because I am successful; I think they are bewitching me. I think they are doing certain things to hinder my progress”* (20 yr old man) (p19) (Okello and Neema, 2007)

Possession by spirits as a cause of depressive symptoms was also more likely to considered when someone's condition was perceived to be chronic, worsening over time or when their presentation included disorderly conduct (Okello and Ekblad, 2006), in which case, spiritual causes were considered or combined with difficult life circumstances as a potential explanatory model (Okello and Neema, 2007). Alternatively, a spiritual cause might be considered when all other explanations made no sense:

*“Because you have money, you don't have misunderstanding with your husband, but then you have thoughts. Then it must be something, an illness. But if money is there and you're are on good terms with your husband and your children are* going *to school; then what kind of thoughts are those? Then they must be an illness, maybe witchcraft or unhappy ancestral spirits”* (FGD, older women)(Okello and Ekblad, 2006)

### 3.4 Consequences

Social isolation, “staying at home” (Familiar et al., 2013) was consistently highlighted as a key impact of the conditions described. Isolation was perceived to be due to withdrawal:

*“too many thoughts detach [separate] you from people. You will be there, you don't even want anyone to call you, when you see someone laughing, you think s/he is laughing at you …”* FGD men (p301) (Okello and Ekblad, 2006)

Isolation was also related to ostracization (among people with severe depression who had been admitted to hospital):

*“since I started falling sick, my friends are no longer close, they keep away. Even those who used to invite me for social activities* like *wedding and funeral rites do not do so any more”* 43 year old woman with recurring depression (p20) (Okello and Neema, 2007)

Links between depression, social isolation and dysfunction in terms of important daily tasks, such as childcare and economic activities and the cyclical nature of these relationships were recognised (Okello and Ekblad, 2006; Okello et al., 2012; Familiar et al., 2013; Petersen et al., 2013; Murray et al., 2017):figure.1

**Fig. 1:**
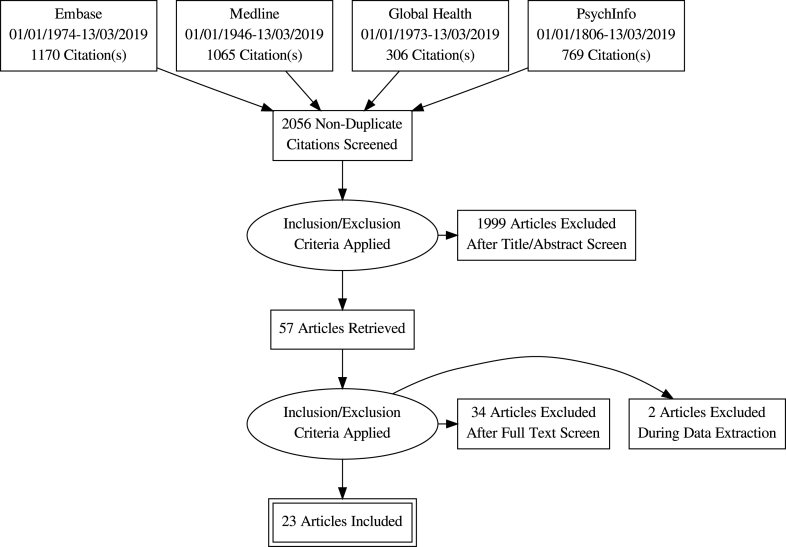
Flow Diagram for Study Selection

“Thinking too much” was highlighted as being problematic, making it difficult to concentrate on daily tasks and causing forgetfulness (Johnson et al., 2017a,b), including missing doses of ART (Kidia et al., 2015) and generally lacking energy to take charge of one's health (Myers et al., 2018). Participants noted adverse effects of their condition upon relationships, particularly with children and partners (Murray et al., 2017). Loss of interest in sex was highlighted as particularly damaging to spousal relationships (Okello and Ekblad, 2006; Okello et al., 2012). For some participants, their illness had resulted in a change in identity and understandings of self:

*“I wasn't as I see me now...sometimes I have got tired. I don't want to be with people. I want to be alone. Sometimes I don't want to talk…I don/t know why I am just like that, because I wasn't like that (before)”* Anele 32 year old woman (p60) (Andersen et al., 2015).

For participants who had been admitted to a psychiatric ward, their illness posed a serious threat to assumptions and plans:

*“I worry* about *my plans for the future, like my plan to go back to school. If the illness does not go away then my hope to go back to school is shattered”* (20 year old man with first episode depression) (p20) (Okello and Neema, 2007)

### 3.5 Social resources and self-help

The therapeutic effects of connection with others were highlighted by study participants from different backgrounds and across settings (Patel et al., 1995a,b; Okello and Ekblad, 2006; Familiar et al., 2013; Petersen et al., 2013; Monteiro and Balogun, 2014; Nakimuli-Mpungu et al., 2014; Irankunda et al., 2017; Johnson et al., 2017a,b; Murray et al., 2017; Myers et al., 2018). Having people with whom it was possible to share problems was integral to the healing and preventative effects of social interaction:

*“To be emotional depressed, I say it depends on the family that you are living with, and how they support you and the friend that you have. When you talk to them, they support you, and you won't be emotional depressed if you get support from your friend and at home.”* (Participant 1, KZN) (p561) (Petersen et al., 2013)

Apart from an immediate social environment that was perceived as supportive, access to people with expertise and understanding of your problems was also recognised as important. For example, talking to elders who could give people living with *nger yec* examples of people with similar problems who had survived their sadness was perceived as helpful by community members in South Sudan (Ventevogel et al., 2013), whilst a trusted older woman or “Senga” (Aunt) was seen as the right person for a younger women to consult about “the stresses of marriage” (Okello and Ekblad, 2006). Being in the company of others, making use of existing mechanisms both for distraction from worries or for problem-solving was encouraged:

*“Some people go to play netball or football. Some go for cultural entertainment like dancing. It is common for women in our area that they sing a lot when they are depressed and they make up their own songs as they do for other family activities”* (mental health worker, Gulu district) (p13) (Nakimuli-Mpungu et al., 2014)

However, Burgess and Campbell noted that advice-seeking and sharing stories supported women to reframe their circumstances, accepting their situation as unalterable. Conceding agency dissipated women’s anger but did not address the social and economic hardship which led to their distress. For example, a woman whose partner had left to live with another wife in a different town commented: “*there is nothing I can do, it is just how men are*” (Burgess and Campbell, 2014).

Drinking and using illicit drugs, seen to be used by some depressed people (men), were activities perceived to have a calming effect in the short-term, but which were viewed negatively (Nakimuli-Mpungu et al., 2014). Helping someone to participate in social activities that were perceived to be normal and healthy was seen as providing a bridge between social withdrawal (“staying inside” (Familiar et al., 2013)) and the community, limiting the extent of social disconnection and the harm it could cause if left to develop (Ventevogel et al., 2013):

*“Maybe you can take him to church and they pray for him..Then when he comes back this way to the majority, he can fit in with people…They counsel him, he gets saved so that he can come back to people and get some freedom.”* (FGD, men) (p305) (Okello and Ekblad, 2006)

Apart from their social aspects, faith and religious activities were perceived to be important therapeutic activities in their own right: engaging in prayer and drawing inspiration from reading scriptures were mentioned as sources of relief (Petersen et al., 2013; Murray et al., 2017).

### 3.6 Formal help-seeking

The coping strategies outlined above were seen as the first steps in addressing depressive illness (Nakimuli-Mpungu et al., 2014). It was recognised that it. was common for people experiencing the problem to stay at home, without intervention from priests, traditional healers or healthcare workers (Familiar et al., 2013; Nakimuli-Mpungu et al., 2014). Choosing from whom to seek help was determined by a combination of perceived severity of symptoms/impact and beliefs about causality. Social networks and religious beliefs shaped the help-seeking pathway (Nakimuli-Mpungu et al., 2014); in four studies consulting a priest or a traditional healer was reported as the next port of call after, or in addition to, sharing problems with a trusted person.

Priests were perceived to impart helpful advice, particularly regarding family matters (Familiar et al., 2013) and were seen to provide spiritual healing, through the mechanism of the person affected attending church or the priest reading “a verse in the bible that can encourage her/him”(Patel et al., 1995a,b; Nakimuli-Mpungu et al., 2014). There was ambiguity about the utility, appropriateness and acceptability of traditional healers among a sample of healthcare workers in Burundi:

*“although [these] traditional approaches are respected and utilised, most key informants (20 out of 23) considered that these practices alone were insufficient to address the symptoms and consequences of mental distress problems”* (p950) (Familiar et al., 2013)

In Uganda, although a majority endorsed a social cause, traditional medicine was preferred by many. Authors speculated that this was due to the diversity of treatment options and skills offered by traditional healers (family conflict resolution, affirmation of cultural beliefs and values, community participation) (Johnson et al., 2017a,b). Also, in Uganda, among people admitted for depression, Okello et al (2007) reported differences in opinion: even among those who agreed on a supernatural cause, some believed that traditional healing was the right way to address an illness believed to be caused by witchcraft, whilst others thought that this was going against Christian doctrine:

*“Even my parents believe that it is the man who is bewitching me. However, my parents are saved and they do not believe in fighting evil powers with evil. Before I was admitted my mother told me, they will take me to church so that they* could *pray for me.* (23 year old woman admitted after attempting suicide)(p21) (Okello and Neema, 2007)

Biomedical treatment was similarly contested. For some, this was a last port of call, when all else had failed or when symptoms became very severe (Patel et al., 1995a,b), for example when people with depression attempted suicide or became loud and aggressive (Familiar et al., 2013). This meant that the problem was often chronic by the time the individual presented at primary care, for example, in Zimbabwe, Patel et al., 1995a,b found that 42% of people had been suffering for more than two years before attending (Patel et al., 1995a,b). Women affected by HIV/AIDS in South Africa had only very limited access to public services (clinicians, social workers), who were perceived to overwhelmed by the burden of problems present in communities (Burgess and Campbell, 2014). In Uganda, a lack of mental health services was identified as a reason for help-seeking elsewhere:

*“Because the* work *of those head doctors isn't spread as yet. Few people know that there are people who can help such a person in western medicine”* (FGD men) (p305-306) (Okello and Ekblad, 2006)

Somatic symptoms sometimes offered a direct route into biomedical services (Patel et al., 1995a,b; Andersen et al., 2015; Myers et al., 2018). Generally, these physical complaints were treated as having a bodily cause and treatment was provided despite a lack of specific diagnosis. For example, pain killers were prescribed for body pain when this was reported to healthcare workers (Aidoo and Harpham, 2001). Nonetheless, sometimes somatic symptoms provided an indirect route to biomedical treatment for depression:

*“I felt fever; I went to the clinic in our village. They gave me some tablets which I took for one week. I did not feel any better so after two weeks they took me to the health centre in Bukomero (subcounty health centre). They checked my blood but they could not find anything. However they gave me a drip and some tablets. I took the tablets for another week. It reduced a little but increased* again *after two weeks. This time my head became chaotic. I started saying things that people could not understand. I was referred to Mulago and my brother brought me”* (18 year old man with first episode of depression) (p20) (Okello and Neema, 2007)

For some, the presentation of the problem negated the suitability of a biomedical approach:

*“It may not be that you have something like fever disturbing you, which you can take to the hospital, but thoughts...Even a friend may not go there because this person has nothing itching her. It is thoughts”*(p295) (Okello and Ekblad, 2006)

*“because* depression *is a pain from the heart...cannot be treated by pills or injections”* (Male) (p54) (Irankunda and Heatherington, 2017)

Help-seeking was flexible and responsive to new evidence, most notably, a lack of improvement or a worsening of symptoms were triggers for a change in approach, sometimes including exchange of incorporation of new ideas about the cause of the problem. Among chronic disease clinic attendees in South Africa, the possible incorporation of depression screening and counselling into treatment models was welcomed by patients, who recognised that “there are many people who have problems. We are talking in the waiting area..” [Participant 11, HIV) for which they did not proactively seek help at health facilities (p1799) (Myers et al., 2018).

## 4 Discussion

For participants, depression was one problem among many, indelibly linked to other life challenges, in terms of its origins, evolution and consequences. Social relationships were the key features in these landscapes. Spousal relationships that were found to be wanting in terms of reciprocal emotional and economic support, sometimes dislocated by HIV, were central to the fabric of descriptions of “burdened hearts”. As has been found to be the case for postnatal depression (Davies et al., 2016), HIV; concerns about disclosure, stigma and the interruption of normal goals and aspirations, was seen to have its own dislocating effect. It is important to note that most study participants were women. Narratives reflect female dependence, immobility and lack of access and control of financial resources, which have been found to play a role in shaping depression elsewhere (Broadhead and Abas, 1998; Patel et al., 2002; Rodrigues et al., 2003; Karasz, 2005; Kazi et al., 2006). Disrupted relationships (with ancestors, family or community members) were the underlying narrative of depressions ascribed a spiritual cause. Consistent with Evans-Pritchard's understanding of witchcraft as a way of making sense of misfortune, where it was given as an explanation those concerned seemed to be seeking to understand why depression had afflicted them, and trying to find a response that was socially and morally satisfying (Evans-Pritchard, 1937). In this context, HIV could be seen to occupy a parallel role to witchcraft, providing a response to the “why me” questions relating to misfortune and possessing moral connotations (Musheke et al., 2013; Vreeman et al., 2015).

As has been noted by others, depression was rarely perceived to reside purely in the body or in the mind but was seen to unfold across both domains (Patel, 1995). This representation of embodied experience as “phenomenological memoir” makes sense as participants struggle to find meaning in their illness (Karasz, 2005; Kazi et al., 2006). Whilst participants looked for coherence between the nature and naming of the problem and their approach to addressing the problem, context actively shaped these explanations. Experiences of living with HIV and HIV-related treatment and care exacted a strong influence on the way in which participants explained their problem, which they saw as an illness, with biomedical models of stress and depression as a framework for their understanding. “Thinking too much”, identified as a key component of depression and anxiety elsewhere (Kaiser et al., 2014), was prominent in descriptions of depression by people living with HIV. This rumination was perhaps activated by the pervasive effect HIV was perceived to have upon individuals lives. Sometimes HIV was cited as a direct cause of illness. These models are supported by healthcare workers, who often consider encouragement of people living with HIV to “live positively” to be part of their role (Patel et al., 2002). Rather than adding an additional layer of stigma, associating depression with HIV provides a framework into which depressive symptoms might be absorbed: when living with HIV, the work of understanding and acceptance of a new self is underway; there is perhaps some evidence of self-efficacy for managing chronic illness, that therefore brings hope for the possibility of resolution of depression (Patel et al., 2002; Rodrigues et al., 2003).

Depression was understood to disrupt natural rhythms: childcare, sex, education, participation in rituals and community events; which also maintain social relationships. Thus, if left to intensify, it threatens to overwhelm and replace the sense of the everyday, and the individual's place in the world that preceded the illness' Ideas about self-help then, were focussed upon preventing dislocation through encouraging social participation, even when this was undesired by the person with the burdened heart. This approach is consistent with social integration theory, originating from Durkheim, which suggests that social support is protective against uncertainty and despair (Berkman et al., 2000).

As others have noted, it is commonplace for people to take a pluralistic approach to dealing with depression, incorporating the different social agencies available to them to address misfortune and illness, to remake the lifeworld (Good, 1994; Patel et al., 1995a,b; William and Healy, 2001). Our findings are consistent with William and Healy's suggestion of “exploratory map” as a more appropriate description of the way in which people actively pursue meaning in their experiences than “explanatory model”(William and Healy, 2001). This description recognises that seeking and making meaning is a process that is characterised by movement and uncertainty, all of which may not be accurately illustrated by an explanatory model, which implicitly assumes stability of the beliefs and conceptualisations described. From the possible explanations available to them participants in the studies included in our synthesis tended to start off with more generalised account of the problem: circumstances that applied to lots of people-poverty, relationship problems. But then if the symptoms were seen to be very severe or did not go away with common sense approaches, they began to think there was an additional problem or, something more specific that required more serious, spiritual or biomedical intervention. Dealing with the problem revolved around addressing alienation, using tangible signs of the shared world, such as religion, to bring the individual back to the familiar. The illness narrative was negotiated, both with family members and healthcare workers, often incorporating elements of their theories. Where priests, social resources and self-help were the selected approach, the process of healing was tangible, explicitly addressing the causes of social dislocation that were understood to be the foundation of depression. Biomedical and traditional approaches to healing are more intangible, dealing as they do with unseen mechanisms. Both therefore occupy a more ambiguous position, commonly called upon as a last resort when more material approaches have failed.

As outlined in the introduction, our approach and search strategy delineated the field of study, supporting a focussed exploration of “depression”, a categorisation determined by the original authors of the studies included in this review. Works that explored “culture-bound syndromes”, mental health in general, beliefs relating to health and illness, systems of healing were therefore not included in our searches. In our view, this directed approach is justified. There is a large literature which demonstrates the validity and resonance of “depression” as a category of human experience. The purpose of this review was therefore to explore how depression is understood and experienced by individuals and communities and how its meaning and impact are constructed, recognising that:

“indigenous psychologies cannot be viewed as hothouse flowers nurtured in isolation, but as alternative modes of being that coexist in a complex global or planetary ecosystem, in which cultural diversity, hybridity and mutual transformations are driven by powerful political and economic forces..” (p11) (Kirmayer et al., 2018)

By focussing attention on the stories individuals tell about depression, we would argue that we add something new to the literature which might have otherwise been overlooked if we had placed a stronger emphasis upon categorisation of experiences and cultural labelling. Our work has something new to say about how experiences of depression affect “one's way of being-in-the-world” in SSA, grounded in the intermingling of concepts of the connectedness of intrapersonal (self), interpersonal (social) and transpersonal (spiritual) worlds with ideas about the importance of actualising personal goals and preferences, the impact of HIV and related stigma upon mental health (Kpanake, 2018). We feel that our broad definition of explanatory models as an inclusion criterion was helpful in ensuring that narratives remained grounded in economic, emotional and relational context, therefore addressing recent critiques of explanatory models that suggest that the methodology has sometimes resulted in a neat narrative of human experience, divorced from meaning (Kleinman, 2013).

As illustrated by the examples described in this synthesis, depression is increasingly named and recognised by healthcare workers and people living in communities in SSA. However, our results demonstrate that this category remains flexible and is attuned to local conceptualisations and expressions. Our description of studies demonstrates the diversity of approaches to exploring explanatory models. The breadth of the work, across several countries and different settings (community-based, people living with HIV, primary care attendees with and without a diagnosis of depression) is both a strength and a limitation, enabling comparisons of conceptualisations but limiting in-depth analysis of any particular contexts. Many population groups are entirely absent; for example, although there are rich data relating to religious self-help and support from leaders, this exclusively refers to the Christian community. The samples are dominated by women. Similarly, the richness and the thickness of the narratives presented varied, and we did not exclude studies on this basis.

Our findings have important implications for global mental health research and practice. It is essential for researchers and practitioners to be fluent in local conceptualisations for detection to be meaningful. Sticking rigidly to DSM or ICD diagnoses will limit detection due to relevance, acceptability of symptoms. Our results suggest that services offered within the health system are likely to be welcomed for particular groups: those who have tried other approaches with little success, those who are already engaged with the health service for other illnesses, e.g. those living with HIV or other chronic illnesses. However, it is unclear from our results how psychological interventions would be perceived within this context. Attendees of health facilities are perhaps more used to receiving medication for their illnesses: talking therapies may potentially be seen as contradictory in this context, particularly when the person may have already participated in similar interactions with priests or traditional healers, without resolution. Carrying out work to understand community perceptions of different models of psychological intervention, including consideration of the legitimacy and power of their mechanisms for effectiveness will be informative (Chowdhary et al., 2016). This work may enhance the acceptability of interventions, stimulating demand and increasing retention in treatment programmes (Patel et al., 2017). Services offered within the health system may thereby become an important option available to people living with depression, alongside other resources available to them in their communities.

However, assuming that it is sufficient to provide interventions designed to address depression in healthcare facilities alone is unrealistic. Mapping community-based resources outside of the health system which people are using to help with their depression should be carried out in preparation for any new interventions. Participants described rich pluralistic systems for addressing depression-like illness problems. These systems included self-help ideas focussed on addressing relationship problems and maintaining or enhancing social support. Global mental health interventions, frequently constituted as stepped care, should take account of these local resources and models. This may require global mental health researchers to change the way we work, working more closely with local communities to develop and evaluate interventions that expand upon the flexibility already offered by stepped care to incorporate social, psychological, biomedical and spiritual dimensions, addressing the person as an interconnected being in the world (Ayorinde et al., 2004; Mkhize and Hook, 2004), by increasing the power of the individuals and institutions people currently turn to when they are faced with depression. This approach is likely to prioritise knowledge derived from different methodologies, such as those used in Participatory Action Research. Our findings suggest that interventions targeting social determinants and addressing the social consequences of depression might be prioritised by communities over conventional clinical outcomes.
